# Detecting diseases in medical prescriptions using data mining methods

**DOI:** 10.1186/s13040-022-00314-w

**Published:** 2022-11-24

**Authors:** Sana Nazari Nezhad, Mohammad H. Zahedi, Elham Farahani

**Affiliations:** 1 Department of Industrial Engineering, K. N. Toosi University of Technology, Tehran, Iran; 2 Sharif University of Technology, Tehran, Iran

**Keywords:** Data mining, Prescription, Prediction

## Abstract

Every year, the health of millions of people around the world is compromised by misdiagnosis, which sometimes could even lead to death. In addition, it entails huge financial costs for patients, insurance companies, and governments. Furthermore, many physicians’ professional life is adversely affected by unintended errors in prescribing medication or misdiagnosing a disease. Our aim in this paper is to use data mining methods to find knowledge in a dataset of medical prescriptions that can be effective in improving the diagnostic process. In this study, using 4 single classification algorithms including decision tree, random forest, simple Bayes, and K-nearest neighbors, the disease and its category were predicted. Then, in order to improve the performance of these algorithms, we used an Ensemble Learning methodology to present our proposed model. In the final step, a number of experiments were performed to compare the performance of different data mining techniques. The final model proposed in this study has an accuracy and kappa score of 62.86% and 0.620 for disease prediction and 74.39% and 0.720 for prediction of the disease category, respectively, which has better performance than other studies in this field.

In general, the results of this study can be used to help maintain the health of patients, and prevent the wastage of the financial resources of patients, insurance companies, and governments. In addition, it can aid physicians and help their careers by providing timely information on diagnostic errors. Finally, these results can be used as a basis for future research in this field.

## Introduction

Studies show that 12 million people worldwide are affected by medical misdiagnosis each year, which means that an average of one in 20 patients is misdiagnosed, with 10 to 20% of those in critical condition. An estimated 40,000 to 80,000 people die each year as a result of these misdiagnoses, with women and minorities typically more affected by between 20 and 30%. In general, 44% of cancers are associated with misdiagnosis, of which the three cancers of prostate, breast, and thyroid have the highest rate of misdiagnosis. 51% of people have encountered a different diagnosis after a breast x-ray when asked for another doctor’s opinion [1].

Studies also show that one-third of medical errors that result in death or disability result from a misdiagnosis or late diagnosis. Misdiagnosis has several complications, the most important of which are unnecessary treatment, increased costs for the patient and the government, physical and emotional stress, and even death [1].

As mentioned, misdiagnosis leads to high costs, for example, the researchers found that diagnostic errors were the leading reason for paid malpractice claims (28.6%) and were responsible for the highest proportion of total payments (35.2%). The researchers estimated that the 2011 inflation-adjusted mean and median per claim payout for diagnostic errors were $386,849 and $213,250, respectively. Also, over 10 years, the amount of compensation paid for diagnostic errors has been $1.8 billion [1].

Improving the diagnostic process is not only possible but also a moral, professional and public health necessity. Therefore, predicting the disease is very important for reducing costs and time overheads and helping the doctor in making decisions. These are the reasons why prescription data can play a vital role in any community to help promote community health [1].

On the other hand, the volume of data is increasing day by day so the need to understand a rich set of data has increased today in all fields including technology, business, and especially medicine. The vast amount of data generated in the medical industry about patients, hospital resources, disease diagnosis, electronic health records, medical equipment, and the like is considered a huge resource that needs to be processed and analyzed in order to save money and to assist physicians in making their decisions [2, 3]. To this end, data mining in the healthcare industry provides a set of tools and methods that can be applied to data to discover hidden patterns in it. The data mining techniques can generally be divided into descriptive and predictive categories. Descriptive methods include clustering and Association rules, and predictive methods include classification and forecasting [3, 4].

Our goal in this study is to use data mining methods to find knowledge in a dataset of medical prescriptions provided by the www.Drugs.com site. By analyzing the prescription drugs for each disease, our proposed method aims to predict the category of each disease and the type of disease that the patient suffers from. Different classification methods have been used to predict diseases based on prescription drugs. Experiments show that the results of the predictions are acceptable. The remainder of this paper is organized as follows: Section 2 deals with the background. The proposed method is explained in Section 3. Section 4 presents the results and the discussions. Section 5 concludes the paper. Finally, Section 6 presents the declarations.

## Background

### Problem statement

Annual misdiagnosis is costly for patients, physicians, insurance companies, and governments. A significant percentage of people around the world incur exorbitant costs due to being prescribed the inappropriate drug, which can, in turn, be the result of misdiagnosis of their disease. The incurred costs, include financial expenses and adverse impact on their health, which in many cases lead to new diseases or even death of the patient. On the other hand, the medical community is not immune to losses resulting from misdiagnoses. A doctor may mistakenly prescribe medication or misdiagnose a disease. This, can lead to disability or even death of a patient, and can negatively affect the progress of the doctor’s career. Following a misdiagnosis, the insurance companies will also incur financial losses by paying the relevant penalty. The fourth entity affected by misdiagnosis is the government, which usually spends huge sums of money annually on importing medicines or allocating capital to drug companies for manufacturing drugs. Especially in recent years, it has been observed that many governments have faced considerable problems due to shortage of a particular drug at some point in time. This can lead to substantial increase in the price of the drug and, in turn, can result in many patients not being adequately treated or even die. On the other hand, through unnecessary import or excess production of some drugs, substantial financial resources may be wasted because the excess drugs have a fixed expiry date and cannot be used thereafter [1, 5–8].

Therefore, providing solutions that can help in the timely detection of drug errors can not only save the lives of many people but can also significantly reduce the cost to patients. It can also be of great help to a large percentage of physicians who will be able to correct their errors in a timely manner. In addition, it can reduce the cost to insurance companies of compensating for misdiagnosis errors. It can also aid the governments’ budgets in the long run. In this way, by providing reliable statistics in a specific time period (for example, 10 years), the amount and type of medication prescribed by doctors for different patients are determined [1].

Hence, predicting the disease is not only important for reducing costs and time overheads and helping the doctors in making decisions, but can also help the governments in numerous fields [1].

### Literature review

In recent years, many studies on the prediction of various diseases, their treatments, and drug discovery have been performed around the world. Different data mining techniques have been used for disease detection and different results have been obtained. The following is a description of these studies for several diseases such as heart disease, diabetes, cancer, etc.

The heart disease has become one of the most common diseases in humans, so today the prediction and diagnosis of cardiovascular diseases at an early stage are necessary in order to reduce mortality from this disease. In recent years, many studies have been conducted in this field, including:

Kondababu et al. (2021) have predicted heart disease using machine learning algorithms. In their study, they discussed many existing methods, among which the proposed HRFLM technique, which uses a combination of random forest (RF) characteristics and linear method (LM), was very accurate with an accuracy level of 88.7% [9]. Jeyaranjani et al. (2021) developed a decision support system based on a supervised learning model for deciding the status of coronary heart disease angiography. The results of their study present the ANN model with 97% accuracy in predicting disease stages. This decision support system helps in early detection [10]. Jothi et al. (2021) proposed a model for predicting heart disease using the decision tree algorithm. In their study, the Decision Tree algorithm can be used on the data set to predict the patient’s risk of heart disease with an accuracy of 81% [11]. Pavithra and Jayalakshmi (2021) proposed a new HRFLC feature selection technique (random forest + AdaBoost + Pearson coefficient). This method helps to predict diseases in a very efficient way and improves the level of accuracy in forecasting [12]. Ramesh et al. (2021) proposed a feature selection algorithm that enhances the performance of any ML approach and is known as Information Gain-based Feature Selection (IGFS). In their study, SVM and RF algorithms showed the highest performance with an accuracy rate of 88% [13]. Maini et al. (2021) proposed a machine learning-based heart disease prediction system for the Indian population. Their proposed system works well for the early detection of cardiovascular disease and can be accessed via the Internet. The best performance RF algorithms have accuracy, sensitivity, and specificity of 93.8, 92.8, and 94.6%, respectively [14]. Kumar and Sahoo (2015) have proposed a new algorithm which combines simple Bayesian and genetic algorithms to improve the classification of heart disease. In this algorithm, classification learns to categorize heart disease datasets into sick or healthy categories. Experimental results obtained from 6 data sets in their study show that the proposed approach is an effective method for classification. Their predictive model assists physicians in the process of efficiently diagnosing heart disease with fewer features [15].

Diabetes is another major medical problem that causes many deaths in the world every year, which is why many studies have been done to predict it, including:

Jain et al. (2021) predicted diabetes using artificial intelligence algorithms on the Pima Indians Diabetes dataset. In their study, the neural network algorithm with 87.88% accuracy achieved the best performance, which can be useful for physicians in the treatment of this disease in its early stages [16]. Kumari et al. (2021) have proposed a soft voting classifier model with a set of three algorithms such as random forest, logistic regression, and simple Bayes to predict diabetic patients. They applied their proposed model to the Pima Indians Diabetes Database and the Breast Cancer Database. Their proposed model has an accuracy of 79.08% in the diabetes dataset and 97.02% in the breast cancer dataset [17]. Khaleel and Al-Bakry (2021) proposed a model that can predict whether a person has diabetes. The results show that the proposed logistic regression with 94% accuracy was more effective in predicting diabetes than other algorithms [18].

Even though there are different data mining classification algorithms for predicting heart disease, there is not enough data to predict heart disease in a diabetic person. Arumugam et al. (2021) adjusted the decision tree model for optimal performance in predicting the chance of heart disease in diabetic patients because it consistently outperformed the simple vector and simple Bayesian models [19].

In today’s world, cancer has become one of the leading causes of death and breast cancer is one of the main causes of death among women worldwide. Therefore, a great deal of research has been conducted in this field, including:

Because early detection and intervention of lymphedema are essential for improving the quality of life of breast cancer survivors, Wei et al. (2021) conducted their study with the aim of developing a symptom warning model for early detection of breast cancer-related lymphedema. Their proposed logistic regression model showed the best performance with AUC = 0.889 (0.840–0.938), sensitivity = 0.771, specificity = 0.883, accuracy = 0.825, and Brier scores = 0.141 and the calibration was acceptable [20]. Dhanya et al. (2020) used existing ensemble techniques along with a combination of supervised machine learning algorithms to develop a new model for predicting breast cancer. Because not all features are necessary to predict breast cancer, and feature selection helps to build an efficient model in such scenarios, they used feature selection techniques. According to the obtained results, it was observed that their proposed stacking ensemble method is an effective and reliable method for predicting breast cancer by f-test feature selection [21]. Onan (2015) has developed a method for creating a cancer diagnosis system that combines the classification of fuzzy-rough nearest neighbors, consistency-based subset evaluation, and fuzzy-rough instance selection technique. This method uses feature selection to improve comprehensibility, shorten training time, and generalize the model. The evaluation results show that the proposed method has 99.71% accuracy and can be used as a reliable tool for automatic diagnosis of breast cancer [22].

In modern times, obesity has become a major threat to health worldwide. Obesity can lead to the development of complex diseases such as stroke, heart disease and liver cancer. Ferdowsy et al. (2021) predicted the risk of obesity using machine learning algorithms. The results show that their proposed logistic regression algorithm has a good performance with 97.09% accuracy [23].

Chronic kidney disease (CKD) is a condition characterized by the gradual loss of kidney function over time. It is usually asymptomatic in its early stages, and early detection is important to reduce future risks. Pinto et al. (2020) used the CRISP-DM method to construct a system that predicts chronic kidney disease conditions. The obtained results show that their proposed J48 algorithm achieved the most suitable result, namely 97.66% accuracy, 96.13% sensitivity, 98.78% specificity and 98.31% precision [24].

Despite long-term efforts to control and prevent medical errors and increase patient safety, medical errors are still one of the leading causes of death in the world, the costs of which attract the attention of policymakers, health care planners and researchers.

Ahsani-Estahbanati et al. (2021) estimate the incidence rate of medical errors both in Iran and worldwide, elicit factors that affect incident rates, estimate the economic burden of medical errors, and outline international and national interventions that can be made to reduce medical errors. Finally, to draw policymakers’ attention to this critical issue, it provides a policy brief related to strategies for dealing with medical errors and associated costs reduction [25].

Today, early diagnosis is a necessity. Malladi et al. (2021) predicted disease through machine learning based on symptoms. According to the results, the CNN algorithm was 84.5% more reliable than the KNN algorithm for predicting a general disease [26].

Dehkordi et al. (2019) predicted what type of physician, public or private, each patient has been referred to and the type of disease he was suffering from. In this study, the dataset includes 70 different types of diseases and 386 different types of drugs and has a total of 600 records. They used a stacking method to improve the prediction model. The results showed that the accuracy for predicting the type of physician was 73.17% and for predicting the type of disease was 57% [27].

Given that data about the prevalence of communicable and non-communicable diseases, as one of the most important categories of epidemiological data, is used for interpreting the health status of communities, Teimouri et al. (2016) calculated the prevalence of outpatient diseases through the characterization of outpatient prescriptions. Among the classification techniques used in this study, the support vector machine with 95.32% accuracy showed the best performance. In the next stage, combining methods are used to improve the results of the individual data mining algorithms. Among these combining methods, Weighted Voting algorithms with an accuracy of 97.16% has the best performance [28].

Trasierras et al. (2022) presented an approach based on emerging pattern mining to analyze cancer through genomic data. Their proposed model includes four different procedures that are specifically designed to deal with RNA-Seq data on cancer. Unlike existing approaches, which are mainly focused on predictive purposes, their proposal aims to improve the understanding of cancer descriptively, not requiring either any prior knowledge or hypothesis to be validated [29].

Frias et al. (2021) improved the prediction of hepatitis C virus outcome using a data mining approach. Their data mining approach identified genetic patterns that escaped detection using conventional statistics. More specifically, the partial decision trees and ensemble models increased the classification accuracy of hepatitis C virus outcome compared with conventional methods [30].

Table 1 compares the above studies.

**Table 1:** Analysis of data mining methods for the above studies

No.	Authors	Year/Title	Journal	Proposed data mining algorithm	Measurement criteria (%)
**1**	Kondababu, A., et al. [9]	2021/A comparative study on machine learning based heart disease prediction	Materials Today: Proceedings	HRFLM (RF + LM)	Accuracy = 88.7%
**2**	Jeyaranjani, J., T. Dhiliphan Rajkumar, and T. Ananth Kumar [10].	2021/Coronary heart disease diagnosis using the efficient ANN model	Materials Today: Proceedings	ANN	Accuracy = 97%
**3**	Jothi, K. Arul, et al. [11]	2021/Heart disease prediction system using machine learning	Materials Today: Proceedings	Decision Tree	Accuracy = 81%
**4**	Pavithra, V., and V. Jayalakshmi [12].	2021/Hybrid feature selection technique for prediction of cardiovascular diseases	Materials Today: Proceedings	HRFLC (RF + ADABOOST + Pearson Coefficient)	–
**5**	Ramesh, G., et al. [13]	2021/Improving the accuracy of heart attack risk prediction based on information gain feature selection technique	Materials Today: Proceedings	SVM و RF	Accuracy = 88%
**6**	Maini, Ekta, et al. [14]	2021/Machine learning--based heart disease prediction system for Indian population: An exploratory study done in South India	Medical Journal Armed Forces India	RF	Accuracy = 93.8% Sensitivity = 92.8% Specificity = 94.6%
**7**	Kumar, Santosh, and G. Sahoo [15].	2015/Classification of heart disease using Naive Bayes and genetic algorithm	Computational Intelligence in Data Mining	Naïve Bayes and Genetic	–
**8**	Jain, Bhavini, et al. [16]	2021/A machine learning perspective: To analyze diabetes	Materials Today: Proceedings	Neural network	Accuracy = 87.88%
**9**	Kumari, Saloni, Deepika Kumar, and Mamta Mittal [17].	2021/An ensemble approach for classification and prediction of diabetes mellitus using soft voting classifier	International Journal of Cognitive Computing in Engineering	Soft voting classifier	Accuracy = 79.08%
**10**	Khaleel, Fayroza Alaa, and Abbas M. Al-Bakry [18].	2021/Diagnosis of diabetes using machine learning algorithms	Materials Today: Proceedings	Logistic regression	Accuracy = 94%
**11**	Arumugam, K., et al. [19]	2021/Multiple disease prediction using Machine learning algorithms	Materials Today: Proceedings	Decision Tree	–
**12**	Wei, Xiaoxia, et al. [20]	2021/Developing and validating a prediction model for lymphedema detection in breast cancer survivors	European Journal of Oncology Nursing	Logistic regression	AUC = 0.889 (0.840–0.938), sensitivity = 0.771, specificity = 0.883, accuracy = 0.825, and Brier scores = 0.141
**13**	Dhanya, R., et al. [21]	2020/F-test feature selection in Stacking ensemble model for breast cancer prediction	Procedia Computer Science	Stacking	–
**14**	Onan, Aytuğ [22].	2015/A fuzzy-rough nearest neighbor classifier combined with consistency-based subset evaluation and instance selection for automated diagnosis of breast cancer	Expert Systems with Applications	fuzzy-rough nearest neighbors, consistency based subset evaluation, and fuzzy-rough instance selection	Accuracy = 99.71%
**15**	Ferdowsy, Faria, et al. [23]	2021/A machine learning approach for obesity risk prediction	Current Research in Behavioral Sciences	Logistic regression	Accuracy = 97.09%
**16**	Pinto, Ana, et al. [24]	2020/Data mining to predict early stage chronic kidney disease	Procedia Computer Science	J48	Accuracy = 97.66% Sensitivity = 96.13% Specificity = 98.78% Precision = 98.31%
**17**	Ahsani-Estahbanati, Ehsan, et al. [25]	2021/Incidence rate and financial burden of medical errors and policy interventions to address them: a multi-method study protocol	Health Serv Outcomes Res Method	Delphi method	–
**18**	Malladi, Ravisankar, Prashanthi Vempaty, and Vyshnavi Pogaku [26].	2021/Advanced machine learning based approach for prediction of skin cancer	Materials Today: Proceedings	CNN	Accuracy = 84.5%
**19**	Dehkordi, Shiva Kazempour, and Hedieh Sajedi [27].	2019/Prediction of disease based on prescription using data mining methods	Health and Technology	Staking	Accuracy (label 1) =73.17% Accuracy (label 2) =57%
**20**	Teimouri, Mehdi, et al. [28]	2016/Detecting Diseases in Medical Prescriptions Using Data Mining Tools and Combining Techniques	Iranian journal of pharmaceutical research: IJPR	Weighted Voting	Accuracy = 97.16%
**21**	Trasierras, Antonio Manuel, José María Luna, and Sebastián Ventura [29].	2022/Improving the understanding of cancer in a descriptive way: An emerging pattern mining-based approach	International Journal of Intelligent Systems	AN APPROACH BASED ON EPM	–
**22**	Frias, Mario, et al. [30]	2021/ Classification Accuracy of Hepatitis C Virus Infection Outcome: Data Mining Approach	Journal of Medical Internet Research	partial decision trees, Ensemble	Sensitivity = 84.3% Specificity = 83.7% AUROC = 0.89

## Methods

### Method: stacking

This model is one type of Ensembles Learning methodology models. The main motivation for developing such a model is to reduce the error rate. The basic assumption of this methodology is that in the Ensemble Learning models the probability of error in identifying the category or position of a new sample is much lower compared to when only one model is employed. Stacking is an Ensemble Learning model that is similar to Boosting and Bagging (Bootstrap aggregation). Boosting is a machine learning group algorithm used to reduce variance and bias. It is based on turning a set of weak learners into strong learners. Due to the fact that the Boosting method focuses on reducing bias, the basic models used in this method are low variance and high bias models. An important method of Boosting is the Adaboost algorithm, which updates the weights attached to each training sample. On the other hand, Bagging is designed to improve the stability and accuracy of machine learning algorithms which are used in statistical classification and regression. Its purpose is to create a hybrid model that is more robust than its base models. Not only does it reduce variance but it also helps prevent overfitting [31].

There are two ways to combine models. The first is voting, in which the predicted class is chosen by the majority of models. The second is Stacking, where the predictions generated by each base model are used as input to a meta-level classifier whose output is the final prediction.

Stacking, sometimes called stacked generalization, is a way to combine several machine learning techniques into one predictive model to improve the predictive accuracy. The main idea of Stacking is to train several different base models and combine them through the training of a meta model which makes the final prediction based on the predictions made by the base models [32]. This is achieved by taking the following steps. First, the available data is used to train all base models. Next, a hybrid model is trained for the final prediction. In this step, the predictions of all base models are used as additional inputs. Stacking has led to good results in both supervised learning techniques such as regression, classification, and distance learning, and also unsupervised learning methods such as neural networks and density estimation. Table 2 compares Bagging, Boosting, and Stacking methods:

**Table 2:** Comparison of three group methods

	Bagging	Boosting	Stacking
**Partitioning data into subset**	Random	Giving misclassified samples higher weight in selection	Various
**Purposes**	Minimizing variance	Increasing predictive ability	Both minimizing variance and increasing predictive ability
**Function to combine models**	Weighted average	Weighted majority vote	A classification method

### The proposed method

In this section, in the first part, the data collection method is explained and then in the second part, a suitable model for predicting the disease and the disease category is presented.

#### Data collection

In this study, we collected medical prescriptions from the www.Drugs.com site, which holds 14,682 records. This dataset includes 1508 diseases and 1615 different drugs. Since a large number of diseases had very few drugs for their treatment and this caused modeling errors, we selected only the diseases that had more than 10 drugs for their treatment as the data set for this study, which includes 5693 records.

Finally, the selected dataset has 719 attributes, which include the name of the disease and the name of the 718 drugs prescribed for the diseases. The selected dataset includes 146 different diseases. These diseases can be considered in 3 general categories as follows:

1. Diseases that are not fatal, such as colds.
2. Diseases that are not usually fatal but can in certain circumstances be fatal, such as sinusitis. Or, diseases that are a risk factor for a fatal disease, such as high cholesterol level that can contribute to a heart attack.
3. Diseases that are often fatal such as pancreatic cancer.

In the next step, we divided the diseases into 22 different categories, with advice from a physician and added a new feature called the disease category to the dataset. Table 3 shows these categories and the diseases that belong to each category.

**Table 3:** Different categories of disease

No.	Categories	Diseases
1	Urology	1. Urinary Incontinence 2. Benign Prostatic Hyperplasia 3. Urinary Tract Infection 4. Kidney Infections (Pyelonephritis)
2	GI (gastrointestinal)	5. Ulcerative Colitis, Active 6. Nausea Vomiting 7. Irritable Bowel Syndrome 8. GERD 9. Erosive Esophagitis 10. Nausea Vomiting, Chemotherapy Induced 11. Crohn’s Disease, Maintenance 12. Inflammatory Bowel Disease 13. *Helicobacter Pylori* Infection 14. Crohn’s Disease, Acute 15. Crohn’s Disease
3	Dermatology	16. Tinea Versicolor 17. Tinea Corporis 18. Pruritus 19. Urticaria 20. Tinea Cruris 21. Keratitis 22. Dermatitis 23. Acne 24. Seborrheic Dermatitis 25. Plaque Psoriasis 26. Pemphigoid 27. Melanoma, Metastatic 28. Bullous Pemphigoid 29. Psoriasis 30. Pemphigus 31. Atopic Dermatitis 32. Eczema
4	Endocrinology	33. Thyroid Cancer
5	General	34. Sarcoidosis 35. Occupational Exposure 36. Nonoccupational Exposure 37. Local Anesthesia 38. Surgical Prophylaxis 39. Pain 40. Anesthesia
6	ENT	41. Rhinorrhea 42. Tonsillitis Pharyngitis 43. Sinusitis 44. Otitis Media
7	Respiratory	45. Pulmonary Hypertension 46. Nosocomial Pneumonia 47. Aspiration Pneumonia 48. Cough 49. Asthma 50. Bronchitis 51. COPD 52. Pneumonia
8	Urology/cancer	53. Prostate Cancer
9	Neurology	54. Parkinson’s Disease 55. Migraine 56. Cluster Headaches 57. Tardive Dyskinesia 58. Narcolepsy 59. Restless Legs Syndrome 60. Muscle Spasm (Involuntary Hypertonicity) 61. Dysautonomia 62Migraine Prevention (Migraine Prophylaxis)
10	Ophthalmology	63. Ophthalmic Surgery 64. Glaucoma, Open Angle 65. Intraocular Hypertension 66. Conjunctivitis, Bacterial 67. Conjunctivitis, Allergic 68. Uveitis
11	Cardiology	69. Obesity 70. Hyperlipoproteinemia Type IIb, Elevated LDL VLDL 71. Hyperlipoproteinemia Type IIa, Elevated LDL 72. High Cholesterol, Familial Heterozygous 73. High Cholesterol 74. Heart Failure (Congestive Heart Failure) 75. Arrhythmia 76. Supraventricular Tachycardia 77. Hypertensive Emergency 78. Hyperlipoproteinemia 79. Atrial Fibrillation 80. Angina 81. Left Ventricular Dysfunction 82. High Blood Pressure (Hypertension) 83. Edema 84. Cardiovascular Risk Reduction
12	Nephrology	85. Nephrotic Syndrome 86. Diabetic Kidney Disease (Diabetic Nephropathy)
13	Infectious disease	87. Influenza 88. Gonococcal Infection, Disseminated 89. Bladder Infection 90. Bacterial Skin Infection 91. Upper Respiratory Tract Infection 92. Tuberculosis, Active 93. Septicemia 94. Hepatitis C 95. Candidemia 96. Bacterial Endocarditis Prevention (Bacterial Endocarditis Prophylaxis) 97. Skin and Structure Infection 98. Peritonitis 99. Endocarditis 100. Bacterial Infection 101. Bacteremia 102. Skin or Soft Tissue Infection 103. Intraabdominal Infection 104. Bone infection (Osteomyelitis) 105. Meningitis
14	Psychology	106. Depression 107. Schizoaffective Disorder 108. Panic Disorder 109. Somatoform Pain Disorder 110. Insomnia 111. Posttraumatic Stress Disorder 112. Bipolar Disorder 113. Obsessive Compulsive Disorder 114. Anxiety 115. Borderline Personality Disorder
15	GI/Endocrinology	116. Cystic Fibrosis
16	GI/cancer	117. Colorectal Cancer
17	Ob-Gyn/cancer	118. Cervical Cancer 119. Breast Cancer, Metastatic 120. Ovarian Cancer
18	Rheumatology	121. Ankylosing Spondylitis 122. Rheumatoid Arthritis 123. Raynaud’s Syndrome 124. Osteoarthritis 125. Osteoporosis 126. Juvenile Rheumatoid Arthritis 127. Psoriatic Arthritis 128. Fibromyalgia
19	Hematology-Oncology/cancer	129. Acute Lymphoblastic Leukemia 130. Lymphoma 131. Breast Cancer, Palliative 132. Breast Cancer 133. Mantle Cell Lymphoma
20	Ob-Gyn	134. Premenstrual Dysphoric Disorder 135. Hot Flashes 136. Vulvodynia
21	Hematology	137. Idiopathic Thrombocytopenic Purpura 138. Febrile Neutropenia
22	Hematology/oncology	139. Chronic Lymphocytic Leukemia 140. Renal Cell Carcinoma 141. Non-Hodgkin’s Lymphoma 142. Hodgkin’s Lymphoma 143. Chronic Myelogenous Leukemia 144. Cancer 145. Acute Myeloid Leukemia 146. Pancreatic Cancer

The first, more important, goal of this research is to predict the type of disease that each sample suffers from. The second goal is to predict which of the 22 different disease categories each sample (representing a patient) belongs to. A group of physicians were tasked with predicting each patient’s disease only by the names of the drugs given to each sample in the dataset. Their final predictions were 100% correct because the results were reviewed three times by each physician. The number of classes (i.e., number of different diseases + other diseases) is 147. Therefore, each sample in the dataset can be labeled by one of 146 different diseases plus other diseases (Altogether, 147 different classes). The purpose of adding other diseases is that if there is a patient whose disease is not one of the 146 diseases listed in Table 3, then it will be included in this category.

Finally, our database contains 720 different attributes, the first attribute is the first label or the name of the disease, the second attribute is the second label or the name of the disease category, and the attribute 3 to 720 are the names of prescription drugs. Each record is a prescription for the patient. In each record, if a drug is prescribed to the patient, this is indicated by 1, and if it is not prescribed, this is shown as − 1. Also, if it is not clear whether this drug is prescribed or not, due to the doctor’s handwriting being illegible, this is recorded as 0. Table 4 shows the final dataset and describes the attributes used in the dataset.

**Table 4:** Attributes description of the dataset

Attribute No.	Attribute	Description	Values
1	Label 1	Disease	147 different values
2	Label 2	Category of disease	22 different values
3–720	Name of Drug	718 different drug names	Each drug prescribed by the doctor for the disease was given a value of 1. The value −1 was assigned to the drug if it was not prescribed, and 0 was assigned if it cannot be determined whether the drug was prescribed (due to the illegibility of the doctor’s handwriting).

#### Modeling

Data mining methods generally fall into two categories: descriptive and predictive [4, 33]. In this paper, 4 predictive methods are used to predict two different labels, described in the previous section. These 4 methods are decision tree, simple Bayes, random forest, and K-nearest neighbors. In the discussion section, we compare the accuracy of these algorithms.

Furthermore, in this section, the Stacking model, which is designed to achieve the highest accuracy, will be described. As shown in Table 4, each sample has many attributes. As a result, there are too many pairwise correlations between the attributes that need to be considered, and this has a negative impact on the accuracy of the prediction model. In addition, overfitting can occur. To prevent overfitting, ensemble learning methods are used, which is one of the most common solutions. In addition, principal component analysis (PCA) on input features was utilized to eliminate the correlation between variables as well as to remove low-value dataset features. PCA is a Dimensionality Reduction method that uses orthogonal linear projections to reduce the number of parameters. In other words, a set of correlated variables is transferred to a new set of non-correlated variables. In general, a vector transformation is used for dimensionality reduction of large datasets.

##### Model

**Fig. 1:**
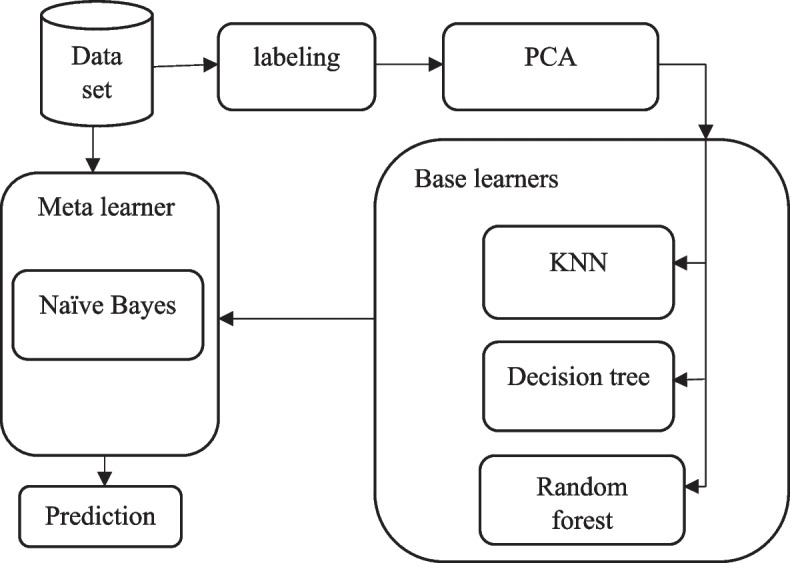
Stacking model

In this section, we present our proposed stacking model for predicting disease categories as well as disease types based on patient prescriptions. This model has three base learners, namely KNN, decision tree and random forest. Naïve Bayes is the Meta learner of this model. Figure 1 shows our proposed Stacking model. In this model, meta features, which are the results of the prediction of the three base classifiers, are added to the original features of the instances. Consequently, the Meta learner, which is the Naïve Bayes (NB) classifier, models the instances with 719 + 3 features. After using PCA, the maximum number of components that generated the best result was 124 features.

## Discussion

The dataset contains 5693 patient records. Two attributes were considered as labels for each record, namely, the disease and the disease category that the patient suffers from. Separate experiments were undertaken on each attribute. The RapidMiner data mining tool is used for performing the experiments.

We first predicted both labels using four single classification algorithms: decision tree, simple Bayes, random forest, and K-nearest neighbors. Tables 5 and 6 compare the accuracy and their kappa score.

**Table 5:** Comparison of single classifiers in predicting label 1

Classification techniques	Accuracy	Kappa score
KNN	58.95%	0.586
Decision Tree	58.67%	0.581
Random Forest	56.68%	0.564
Naïve Bayes	59.09%	0.585

**Table 6:** Comparison of single classifiers in predicting label 2

Classification techniques	Accuracy	Kappa score
KNN	67.81%	0.657
Decision Tree	67.61%	0.649
Random Forest	65.24%	0.618
Naïve Bayes	68.73%	0.657

According to Tables 5 and 6, it can be seen that the simple Bayesian algorithm has better performance for both labels than other algorithms. However, as we said in the modeling section, to improve the prediction model, we propose an Ensemble Learning model.

The parameters of base learners and their values for the proposed model are shown in Tables 7 and 8.

**Table 7:** Accuracy of the stacking model in predicting Label 1

Base learner / Meta Learner	KNN	Decision Tree	Random Forest	Naïve Bayes (Meta Learner)
**Parameters**	-K = 3 - Weighted vote	-With pre-pruning -Maximal depth = 10	-Maximal depth = 10 -Number of trees = 62 -Without pre-pruning	-Laplace correction
**Accuracy**	**62.86%**			
**Kappa score**	**0.620**

**Table 8:** Accuracy of the stacking model in predicting Label 2

Base learner / Meta Learner	KNN	Decision Tree	Random Forest	Naïve Bayes
**Parameters**	-K = 3 - Weighted vote	-With pre-pruning -Maximal depth = 7	-Maximal depth = 10 -Number of trees = 54 -Without pre-pruning	-Laplace correction
**Accuracy**	**74.39%**			
**Kappa score**	**0.720**			

Table 9 shows that the accuracy of the other ensemble methods for the first and second labels of the dataset is less than the stacking method. The learner is a decision tree for both Adaboost and Bagging methods, and its parameters are shown in Table 9.

**Table 9:** Comparison of other ensemble techniques

Classification Techniques	Parameters of learner	Accuracy	Kappa score
**Adaboost (Label 1)**	- Maximal depth = 10 - Number of preprunning = 3	57.14%	0.562
**Bagging (Label 1)**	- Maximal depth = 6 - Number of preprunning = 3	51.43%	0.505
**Adaboost (Label 2)**	- Maximal depth = 7 - Number of preprunning = 3	64.56%	0.613
**Bagging (Label 2)**	- Maximal depth = 7 - Number of preprunning = 3	64.21%	0.611

It should be noted that the results of the experiments would have been worse if PCA had not been used. In fact, the use of PCA has significantly improved the results. Table 10 shows the accuracy of the proposed stacking model without applying PCA.

**Table 10:** Accuracies without applying PCA

Stacking Model	Accuracy	Kappa score
**Stacking Model for label 1**	56.68%	0.564
**Stacking Model for label 2**	70.89%	0.684

Tables 11 and 12 each compares the accuracy of the stacked model for predicting the values of label 1 and label 2, respectively. The models utilize various base learners with optimum parameter values, and different meta learners. This led to the best three results after the stacked model in Tables 7 and 8 for each model.

**Table 11:** Evaluation of best classifiers for label 1 of the dataset

Base learner/ Meta learner	Decision tree, Random Forest, Naïve Bayes / KNN	Random Forest, Naïve Bayes, KNN / Decision tree	Decision tree, Naïve Bayes, KNN / Random Forest
**Parameters**	- Maximal depth = 10 - Number of trees = 62 - Laplace correction - K = 6	- Number of trees = 62 - Laplace correction - K = 3 - Maximal depth = 10	- Maximal depth = 10 - Laplace correction - K = 9 - Number of trees = 54
**Accuracy**	54.29%	57.14%	61.60%
**Kappa score**	0.532	0.562	0.613

**Table 12:** Evaluation of best classifiers for label 2 of the dataset

Base learner/ Meta learner	Decision tree, Random Forest, Naïve Bayes / KNN	Random Forest, Naïve Bayes, KNN / Decision tree	Decision tree, Naïve Bayes, KNN / Random Forest
**Parameters**	- Maximal depth = 7 - Number of trees = 54 - Laplace correction - K = 3	- Number of trees = 54 - Laplace correction - K = 3 - Maximal depth = 10	- Maximal depth = 10 - Laplace correction - K = 9 - Number of trees = 54
**Accuracy**	72.98%	56.14%	70.68%
**Kappa score**	0.710	0.525	0.678

Eventually, according to the results obtained above and comparing them with the results of Tables 7 and 8, the Stacking model of Tables 7 and 8 with an accuracy of 62.86% for the first label and 74.39% for the second label and a kappa score of 0.620 for the first label and 0.720 for the second label had the best results for both labels and was selected as the final model. This is summarized in Table 13 for accuracy and Kappa score.

**Table 13:** Accuracy and kappa score of the final stacking model

Final Model	Accuracy	Kappa score
**Stacking model for predicting Label 1**	62.86%	0.620
**Stacking model for predicting Label 2**	74.39%	0.720

Our proposed model is more accurate than the model presented in a similar study conducted by Dehkordi et al. (2019). Their model for disease prediction has an accuracy of 57% while our proposed model has an accuracy of 62.86%.

It should be noted that the database used in our study is larger than the database in the study of Dehkordi et al. (2019). Our database consists of 146 different diseases and 718 different drugs, while the database used in their study includes 70 different diseases and 386 different drugs.

Finally, Tables 14 and 15 are provided to demonstrate that our proposed ensemble method has higher accuracy and kappa scores compared to single classifiers such as KNN, Naïve Bayes, decision tree, and random forest for the first and second label of the dataset. According to Section 4, an Ensemble Learning model provides the opportunity to reach a better result.

**Table 14:** Comparison of single classifiers in predicting label 1

Classification techniques	Accuracy	Kappa score
KNN	58.95%	0.586
Decision Tree	58.67%	0.581
Random Forest	56.68%	0.564
Naïve Bayes	59.09%	0.585
Stacking	62.86%	0.620

**Table 15:** Comparison of single classifiers in predicting label 2

Classification techniques	Accuracy	Kappa score
KNN	67.81%	0.657
Decision Tree	67.61%	0.649
Random Forest	65.24%	0.618
Naïve Bayes	68.73%	0.657
Stacking	74.39%	0.720

Therefore, considering the acceptable level of accuracy and kappa score obtained from this study, this model can be used to help solve the problems mentioned in the problem statement section and can be adopted as the proposed model to improve the diagnosis process.

If the use of this proposed model becomes widespread, the Ministry of Health can determine the type and amount of drug use by knowing the statistics and the number of patients who have used this model over a long period (for example, 5 to 10 years). By knowing this statistic, the annual need for each drug in the country can be more accurately estimated, and consequently, it will be possible to procure the sufficient amount of each medicine in advance and avoid overspending.

Also, the healthcare industry can use this information to identify people with chronic illnesses, which is an indirect way makes it possible to estimate the prevalence of such illnesses.

Our next goal in research is to provide a website or mobile application using the model proposed in this article. This allows the patients to search the drugs prescribed by a doctor to find out whether these drugs are related to their disease according to our proposed model. If a discrepancy is detected by the patient, he can seek further advice from his doctor again. The advantage of using such a website or application is that the information about a wide range of diseases and related medications can be collected together. In addition, each user can save their medical history by having a unique account. This is important because, in some countries where there is no electronic health record, our proposed website/application can be used to create such a record for each person in the community, using the medical records available in insurance companies and the drugs prescribed by physicians.

## Conclusion

According to Johns Hopkins University of Medical Sciences’ research on medical diagnosis, misdiagnosis is at the forefront of serious medical errors. Most people will likely experience at least one misdiagnosis in their lifetime, which can sometimes have devastating consequences. Also, this issue has an indirect adverse impact on the professional life of doctors and the quality of services provided by them, Furthermore, the complaints raised as a result of such errors have always been one of the most important stressors for doctors. Hence, providing solutions to help improve the correct prediction of the disease and the correct administration of the drug is very important and can be the first step in treatment of a patient. The overall purpose of this paper was to predict what kind of disease, from 147 different classes, each patient suffers from and to which of 22 separate categories each disease belongs. Four data mining classification algorithms were used: decision tree, random forest, Naïve Bayes, and KNN. Then a stacking model was used to improve the performance of the algorithms and achieve optimal results. This proposed model had better performance than the individual classifiers by showing the accuracy and kappa score of 62.86% and 0.620 respectively for disease prediction and the accuracy and kappa score of 74.39% and 0.720 respectively for predicting the category of disease, so it was presented as the final model. In the dataset, three different base learners namely KNN, decision tree, and random forest were applied for classifying in the Stacking operator. The results of our final model can help patients, physicians and medical staff, insurance companies as well as governments.

The results of this study can be used as a platform for future research in this field. We suggest to other interested researchers to study other drugs and diseases as input features. In addition, other data mining techniques such as Association Rules can be used. Association rules are in the group of descriptive methods, so only some descriptive rules can be derived from the dataset.

## Data Availability

The datasets generated and/or analyzed during the current study are available in the [Drugs] repository, [https://www.drugs.com/].

## Abbreviations

KNN: K-Nearest Neighbors
PCA: Principal Component Analysis
